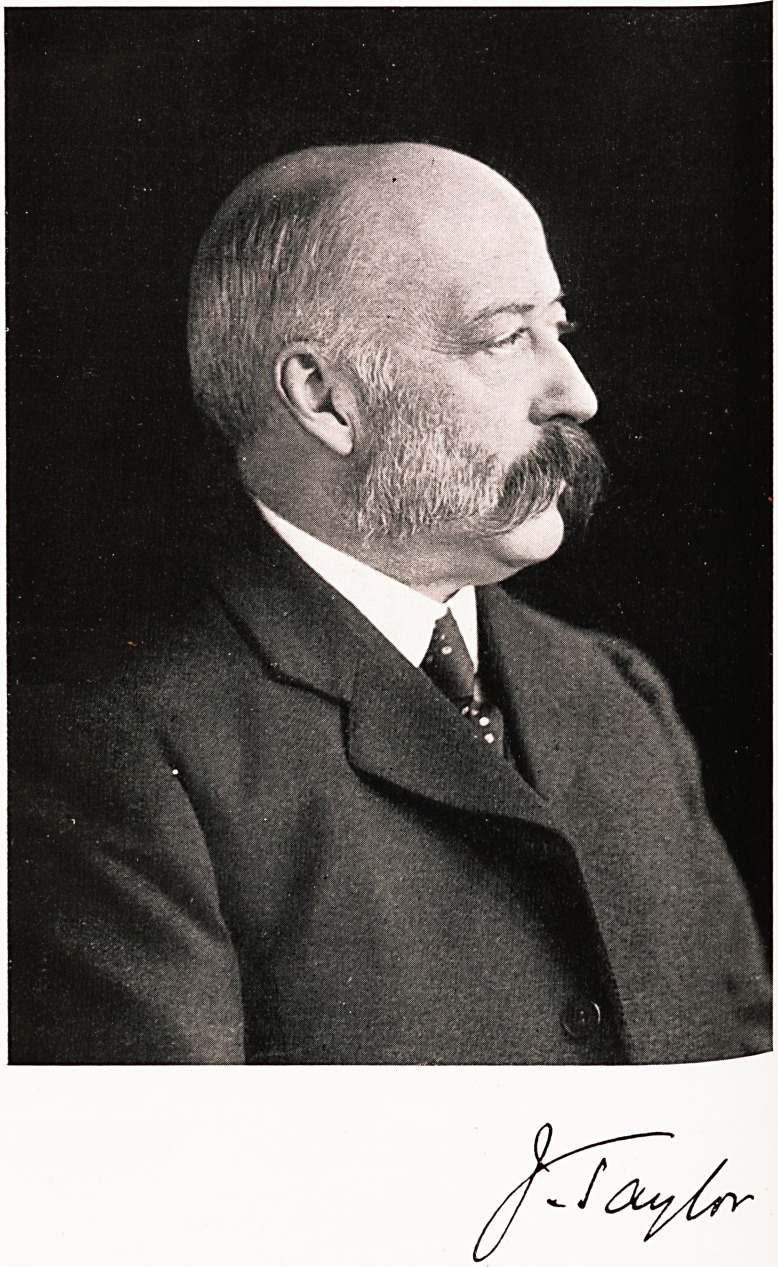# James Taylor

**Published:** 1925

**Authors:** 


### Obituary.

### JAMES TAYLOR.

Many are those who feel deeply the loss of a good friend and valued colleague by the death of James Taylor on March 22nd, at the age of seventy-one. His father, who was in practice in Ashley Road, sent James, the elder of his two sons, to the Bristol Medical School, who after qualifying by taking the M.R.C.S. proceeded to Edinburgh University in 1874 for further study, with a view to taking his degree, but unfortunately his father's death in 1876 compelled his return to Bristol to take on his father's practice. He continued to live with his mother at Wellington House, Ashley Road, up to the time of her death, when he removed to his house in Alma Road.

During nine years he rendered good service to this Journal as the Editorial Secretary, and in 1906 was elected President of our Medico-Chirurgical Society.

James Taylor was one of the earliest pioneers in Röntgen ray work, towards the development of which his previous experience in photography was very helpful. Even eighteen years ago his skiagrams of conditions involving special difficulties, such as nasal sinuses and particularly his stereoscopic plates, were among the first successful examples, in this country, at any rate. When one recalls the relatively crude results obtained at the outset, and how little was foreseen of the later wide range of the sphere of usefulness, and the highly-developed technic that evolved, we realise how greatly medical progress in relation to this aspect of scientific work was indebted to Taylor, who had to depend largely on his own initiative and patient perseverance. He founded the Skiagraphic Department at the Bristol Royal Infirmary in 1898, and took an active share in the development of X-ray therapy, contributing an able article on "X-ray Therapeutics" to this Journal in 1906.

On the outbreak of the Great War Taylor, who held a Captain's commission, R.A.M.C.T., was placed in charge of the skiagraphic work at the Second Southern General Hospital, where his untiring and devoted work was invaluable, and was continued at the Royal Infirmary till he resigned in 1918 the charge of the department which he had held for twenty years.

Taylor inherited a love of music from his father, and for a long period he and his double-bass were an essential feature of most Clifton amateur concerts, and more particularly he constantly assisted in the performances of the Clifton Amateur Operatic Society and the Medical Dramatic Club, until his health began to fail. He was devoted to golf, and furthermore was a keen Mason, being a Past Master of St. Vincent Lodge, in which he was initiated in 1898. He further was appointed Provincial Grand Organist in 1904.

The appreciation of our late colleague in the *Bristol Times and Mirror* so truly reflects the man that we cannot refrain from quoting from it:

"He never came prominently before the public, yet he had a host of friends, and was particularly popular among his professional brethren. This may be attributed to the fact that he was a man of wide interests and keen about many things outside medicine. His patients really loved him, and his kindly, gracious manner often did them as much good as the treatment they received at his hands. Of late years their number greatly diminished, and became a mere handful, because, without definitely retiring from practice, physical infirmities restricted his activities, and he gave up attending all save those whom he had known all their lives. To them he ministered with his customary care and patience, though it obviously became a labour to go his daily rounds."

He was a bachelor and had lost by death every near relative, his only brother, Frank, who had practised in Redland, having died of tuberculosis in 1886 at the age of twenty-nine; but his memory will long be cherished by colleagues and friends, by all of whom he was greatly beloved.

BIBLIOGRAPHY.

"The X-ray History of a Fracture," *Bristol M.-Chir. J.*, 1900, xviii. 214.

"The use of X-rays in the Diagnosis of Renal Calculi," *Bristol M.-Chir. J.*, 1902, xx. 44.

"X-ray Therapeutics," *Bristol M.-Chir. J.*, 1906, xxiv. 289.

## Figures and Tables

**Figure f1:**